# Effects of omega 3 supplementation in elderly patients with acute myocardial infarction: design of a prospective randomized placebo controlled study

**DOI:** 10.1186/1471-2318-14-74

**Published:** 2014-06-13

**Authors:** Kristian Laake, Peder Myhre, Linn M Nordby, Ingebjørg Seljeflot, Michael Abdelnoor, Pål Smith, Arnljot Tveit, Harald Arnesen, Svein Solheim

**Affiliations:** 1 Center for Clinical Heart Research, Oslo University Hospital, Ullevål, Oslo, Norway; 2 Department of Cardiology, Akershus University Hospital HF, Lørenskog, Norway; 3 Department of Cardiology, Oslo University Hospital, Ullevål, Oslo, Norway; 4 Faculty of Medicine, University of Oslo, Oslo, Norway; 5 Center of Clinical Research, Unit of Epidemiology and Biostatistics, Oslo University Hospital, Ullevål, Oslo, Norway; 6 Department of Medical Research, Vestre Viken Hospital Trust, Bærum Hospital, Rud, Norway

**Keywords:** Omega 3 fatty acids, Acute myocardial infarction, Randomized placebo controlled trial, Elderly

## Abstract

**Background:**

Both epidemiological and randomized clinical studies suggest that supplementation with very-long-chain marine polyunsaturated n-3 fatty acids (n-3 PUFA) have cardioprotective effects, however these results are not without controversy. Study population, sample-size, type of supplementation and type of endpoint have all varied widely accross different studies.

Therefore, the aims of the present study are to evaluate the effect of 2 years supplementation with capsules of very-long chain marine n-3 PUFA on top of standard therapy in elderly patients after acute myocardial infarction (AMI).

In addition, special characteristics of this population with regard to prediction of clinical outcome will be investigated. The hypothesis is that this supplementation on top of modern therapy will reduce the occurence of major cardiovascular events (MACE). We present the design of the OMEMI (OMega-3 fatty acids in Elderly patients with Myocardial Infarction) study.

**Methods/Design:**

The OMEMI study is designed as a randomized, placebo-controlled double-blind multicenter trial.

Included are patients ≥70-82 years of age who have sustained AMI. Patients of either gender are eligible. Sample size calculation based on existing literature has resulted in the need for 1400 patients followed for 2 years, based on the assumption that the n-3 PUFA supplementation will reduce MACE with 30%. The study medication is Pikasol® Axellus AS, Norway, 3 capsules (1.8 g eicosapentaenoic acid (EPA) + docohexaenoic acid (DHA)) per day, and matching placebo is corn oil. The Primary end-point is the composite of total mortality, first non-fatal recurring AMI, stroke and revascularization. Secondary end-point is the occurrence of new onset atrial fibrillation. Extensive biobanking will be performed, including adipose tissue biopsies. Compliance will be assessed by measurements of the fatty acid profile in serum, sampled at inclusion, after 12 months and at the end of study.

**Discussion:**

The OMEMI study is scheduled to terminate when the last included patient has been followed for 2 years. To the best of our knowledge, the OMEMI study is the first to evaluate the effect of n-3 PUFAs on CVDs and mortality in a high risk elderly population having suffered an acute myocardial infarction.

**Trial registration:**

ClinicalTrials.gov, NCT01841944

## Background

Acute myocardial infarction (AMI) is a major cause of mortality and morbidity in westernized countries [1,2]. With a mean age of individuals suffering an AMI of about 72 years, a major proportion of the patients are above 70 years [1-3]. Although the management of AMI has improved, still a significant number of patients, especially elderly, are at increased risk for adverse events.

The possible benefit of marine omega-3 polyunsaturated fatty acids (n-3 PUFAs) in the prevention of atherosclerosis, the main underlying process behind coronary heart disease (CHD) and an AMI, was first proposed by Dyerberg & Bang after their studies on the Greenland Eskimos in the 1970s [4-6].

Later, a considerable amount of research has been performed on the subject, and n-3 PUFAs have been shown, in some studies, to reduce morbidity and mortality in patients with various cardiovascular disease (CVD) states [7-11], also in elderly individuals [12]. In the DART trial [13], Burr et al. showed that 2033 post-AMI patients randomised to receive or not receive advice on dietary components, those adviced to eat fatty fish had a 29% reduction in 2 year all-cause mortality compared with those not adviced. In the GISSI-4 study [7] 1 g n-3 PUFA/day reduced sudden cardiac death by 40% after 3 years in patients after an AMI [8], and in the DOIT study [14], although not statistically powered for clinical end-points, a 50% reduction in mortality with supplementation of 2.4 g PUFAs/day was achieved after 3 years in elderly patients at high risk for CVD. In a more recent study, higher plasma levels of n-3 PUFAs in 2692 healthy older adults were associated with lower total mortality, especially CHD deaths [15].

However, data is still inconsistent. Recent clinical trials and meta-analyses on n-3 PUFA and CVD, have suggested limited effect [16-19], but the studies differ widely in dosage of study medication, population size, participants disease states and time of follow-up. The lack of effect might also be attributed to the state of art drug treatment not available in the older trials [20]. All these factors may contribute to the confusion in the field. Both the SU.FOL.OM3 [16] and OMEGA trial [17] are considered statistically underpowered [21]. The even larger Alpha Omega trial [18] with 4837 participants clinically diagnosed with myocardial infarction up to 10 years before randomization, showing no effects of n-3 PUFAs on CVD, has also been critized for being underpowered, for using low doses omega-3 and for the formulation used which may nullify any benificial effect [22]. The lack of unanimous agreement is typically shown in the 2 recent meta-analysis by Rizos et al. and Delgado-Lista et al., reviewing n-3 PUFAs effect on CVD including many of the same studies, but coming to opposite conclusions [23,24].

Results from studies on mechanisms have shown that the cardioprotective effects of n-3 PUFA acts through a range of processes involving triglyceride lowering, antiinflammatory, antiarrhythmic and antithrombotic effects in addition to a beneficial influence on blood pressure, heart rate and endothelial function [25,26].

## Methods/Design

The OMEMI (OMega-3 fatty acids in Elderly patients with Myocardial Infarction) study is a prospective, randomized, placebo-controlled, double blinded multicenter trial.

### Aim of study

The aim of the study is to investigate the effects of supplementation with 1.8 g/day of n-3 PUFAs on cardiovascular morbidity and mortality during a follow-up period of 2 years in an elderly population after having experienced an AMI. In addition, special attention to the characteristics of this elderly population in general and with regard to prediction for clinical outcome will be investigated. An extensive biobank will be established, including adipose tissue samples as well as serum for specific fatty acid analysis. Our hypothesis is that supplementation of n-3 PUFAs will reduce the risk for cardiovascular events.

### Patients and centres

Patients will be recruited from three hospitals in the Oslo area, Oslo University Hospital (OUS Ullevål) (study center), Akershus University Hospital (AHUH) and Vestre Viken Bærum Hospital (VVBH). All patients with a diagnosis of AMI, being ≥70-82 years of age will be screened at the respective wards, and eligible patients will be asked for participation after oral and written information will be given. In accordance with the Declaration of Helsinki [27], written consent is obtained. Inclusion will be within 2–8 weeks after the acute myocardial infarction. The inclusion and exclusion criteria are given in Table 1. Permuted block randomization will be undertaken by use of consecutively numbered non-translucent envelopes containing allocation message to either of the randomized groups in a 1:1 ratio, arranged by the Unit of Epidemiology and Biostatistics, OUS, according to tables of random numbers, stratified by centers (OUS/AHUH and BH).

**Table 1:** Inclusion and exclusion criteria in the OMEMI trial

***Inclusion criteria***	***Exclusion criteria***
- Diagnosis of acute AMI (type I,II,IV) according to current guidelines.	- Documented intolerance for Omega-3 fatty acids.
- Age 70–82, either gender.	- Comorbidity thought to be incompatible with compliance to study drugs.
- Not being part of another randomized trial.	- Comorbidity thought to reduce survival for the follow-up time of 2 years.
- Understand Norwegian and giving written consent to participate.	

### Drug regimens and treatment strategies

Patients will be treated according to general guidelines and continue standard medication during the study period. At inclusion the patients will be randomized to either n-3 PUFA or placebo. The patients randomized to the active treatment, will receive Pikasol®, 3 capsules (1.8 g EPA + DHA)/day, and those in the placebo group will receive 3 identically designed capsules of corn oil. Corn oil has been used as placebo in many randomized studies on n-3 PUFAs in CVD. It is considered safe and without any known adverse effects or influence on atherothrombosis. Patients being on supplementation with n-3 fatty acids at inclusion will be permitted to continue with a daily dose of 1 child-spoon with cod-liver oil, but not with capsules containing n-3 fatty acids beyond the study drugs. The study drugs will be provided by Axellus AS (Oslo, Norway) and picked up by the participant in sealed containers from the respective hospital pharmacies at OUS and AHUH at each visit.

### Study visits

**Figure 1:**
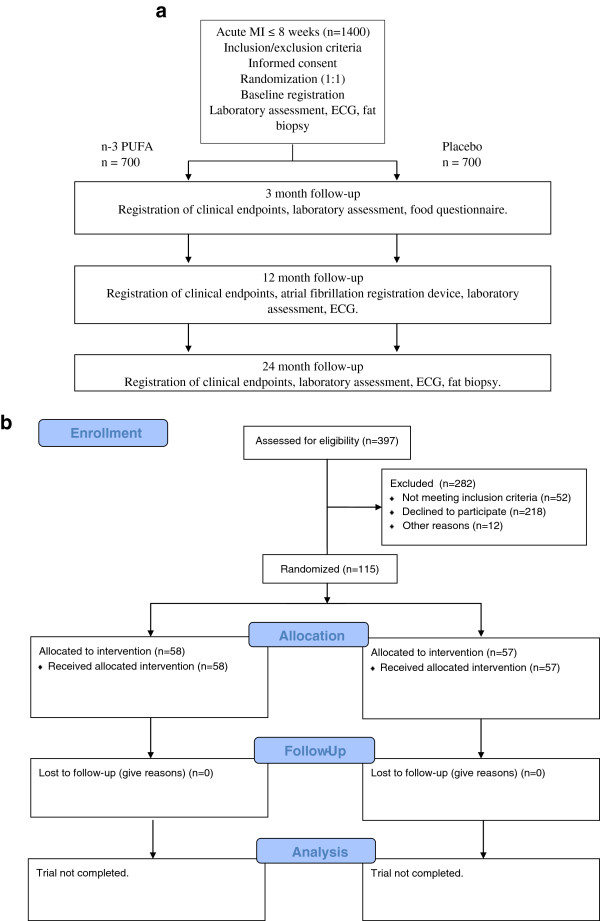
Design (a) and flow-chart according to CONSORT (b) in the OMEMI trial.

The study design of the OMEMI trial is visualized in Figure 1A. The flow-chart of the OMEMI trial according to CONSORT 2010 (checklist in additional file 1) is visualized in Figure 1B. Baseline registration include medical history, use of medication and clinical examination. Participants will also be asked to fill out a food questionaire. ECG will be recorded at baseline, and at 12 and 24 months and a biobank will be established at all time points including fat biopsies at baseline and end of study.

Compliance will be asessed by measurements of the fatty acid profile in serum sampled at inclusion, 12 months and at end of study. The patients will be followed up for two years. Patients that for any reason have been off study medication for more than 4 consecutive weeks will be classified as drop-outs.

### End points

The primary endpoint of the OMEMI study is the composite of total mortality, first event of non-fatal AMI, stroke and revascularization (MACE). Secondary endpoint is occurrence of new onset atrial fibrillation, registered by ECG at follow-ups, and from any hospital admissions during the study period. In addition, patients will be equipped with an electronic device manufactured by Zenicor Medical Systems AB (Stockholm, Sweden) at 1 year follow-up, which allow twice daily ECG recordings at home for 2 weeks of any occurence of atrial fibrillation.

Among patients who are not able to attend the last visit, the clinical end-points will be recorded by the investigator on request. Cause-specific mortality data will be obtained from death certificates provided from the Database of Statistics, Norway. Evaluation of end-points will be undertaken by an external End-point Committee. Internationally accepted diagnostic criteria will be used.

### Substudies

Extensive biobanking will be undertaken according to accepted common sampling and processing protocols at all study visits. Samples will be frozen and create the basis for different biochemical substudies:

– Serum fatty acid profile for compliance and correlation analyses

– Special characteristics of this elderly population in general with regard to prediction for clinical outcome

– Antiplatelet therapy in the elderly

– Gender differences

– Effects of PCI/thrombolysis

Effects of n-3 PUFAs in the elderly on:

– Markers of Endothelial function; Inflammation; Coagulation and Fibrinolytic activity, measured as circulating proteins and mRNA in leukocytes

– Expression of relevant genes associated with atherothrombosis in adipose tissue

– Influence of genetic polymorphisms on atherothrombosis

– Heart rhythm (HRV) by use of a new available method (Zenicor Medical Systems AB)

### Adverse events and monitoring

All adverse events, including gastroenteric complaints and possible interaction with other drugs that are used will be recorded. As a majority of the patients will be treated with antiplatelet drugs, special attention will be given to bleeding complications in this elderly population. Standardized bleeding definitions using the BARC criteria will be used [28]. Regular reports will be delivered to the Data Safety and Monitoring Board (DSMB). Serious adverse events (SAE) will be reported to the National Drug Authority. CIOMS (Council for International Organizations of Medical Sciences) form will be used for reporting SAE.

### Statistical analyses

According to the results from the GISSI-4 and DOIT trials [7,14], we anticipate that the supplementation of n-3 PUFA on top of modern therapy will reduce the combined cardiovascular end-point of death, non-fatal AMI, stroke and revascularizations (MACE) by 30% in the active drug group. Based on data from registries [3,29] and with an alpha of 0.05 and a power of 80%, 611 patients would be needed in each study group. Allowing for an estimated drop-out rate of 10-15%, a total number of 1400 patients will be included. No interim analyses are planned, but the DSMB will have access to the total number of primary end-points for potential prolongation of the study period or increase in the number of participants. Statistical analyses will primarily be performed according to the intention-to-treat principle.

### Approval

The protocol has been approved by the Regional Committee for Medical Research Ethics (ref. number: 2012/1422), and the study has been registered at ClinicalTrials.gov, April 16th 2013, NCT01841944.

### Administrative matters

The study has a Steering committee for monitoring the study progress and quality. All endpoints and SAE will be finally evaluated before closure of the study.

## Discussion

The OMEMI study is scheduled to terminate when the last included patient has been followed for 2 years, probably in 2016/17. Selected baseline characteristics of the first 115 patients included are shown in Table 2. To the best of our knowledge, the OMEMI study is the first to evaluate the effect of n-3 PUFAs on CVDs and mortality in a high risk elderly population having suffered an acute myocardial infarction.

**Table 2:** Baseline characteristics of the first 115 subjects at inclusion in the OMEMI trial; Data presented as percentages or median values (25, 75 percentiles)

Age (y)	75 (72, 78)
Gender (male/female) (%)	73.9/26.1
Current smoker (%)	11.3
BMI (kg/m^2^)	25.5 (23.9, 28.1)
S-total cholesterol (mmol/L)	3.80 (3.20, 4.40)
S-LDL (mmol/L)	2.06 (1.69, 2.52)
S-HDL (mmol/L)	1.26 (1.03, 1.59)
S-triglycerides (mmol/L)	1.15 (0.88, 1.54)
Systolic blood pressure (mmHg)	135 (125, 150)
Pulse rate (bpm)	66 (60, 72)
STEMI (%)	33.9
Troponin-T (peak level) (ng/L)	735 (168, 2562)
Previous atrial fibrillation (%)	13.9
Previous myocardial infarction (n)	46 (40.0%)
Aspirin (%)	93.9
Clopidogrel (%)	40.8
Prasugrel (%)	12.1
Ticagrelor (%)	40.0
Anticoagulation (%)	16.5
Betablocker (%)	89.5
ACE-I/AT II blocker (%)	64.3
Calcium channel blocker (%)	21.7
Statin (%)	96.5
Diuretic (%)	27.8
Nitrates (%)	13.0
n-3 PUFA supplements (%)	47.3

ACE-I/AT II: angiotensin-converting enzyme inhibitors/angiotensin II receptor blockers; S-LDL: low density lipoprotein; S-HDL: high-density lipoprotein; STEMI: ST-segment elevation myocardial infarction.

## Competing interests

The authors declare that they have no competing interests.

## Authors’ contributions

KL conducts the study and is responsible for analysis and interpretation of the data, drafted and revised the manuscript. PM and LMN conducts the study, will contribute to the interpretation of the results and revised the manuscript. MA made contributions to the statistics used, study protocol and design. IS and HA conceived of the study, contributed to the study protocol, obtained funding and discussed the manuscript. PS and AT contributed to the study design and protocol and assists in the data collection. SS coordinates the study and made substantial contribution to the study protocol, will contribute to data analysis and intepretation, and revised the manuscript. All the authors contributed to revision and approval of the final manuscript.

## Pre-publication history

The pre-publication history for this paper can be accessed here:

http://www.biomedcentral.com/1471-2318/14/74/prepub

## Supplementary Material

Additional file 1 CONSORT 2010 checklist of information to include when reporting a randomised trial*. Click here for file
